# Considerations in the development of circulating tumor cell technology for clinical use

**DOI:** 10.1186/1479-5876-10-138

**Published:** 2012-07-02

**Authors:** David R Parkinson, Nicholas Dracopoli, Brenda Gumbs Petty, Carolyn Compton, Massimo Cristofanilli, Albert Deisseroth, Daniel F Hayes, Gordon Kapke, Prasanna Kumar, Jerry SH Lee, Minetta C Liu, Robert McCormack, Stanislaw Mikulski, Larry Nagahara, Klaus Pantel, Sonia Pearson-White, Elizabeth A Punnoose, Lori T Roadcap, Andrew E Schade, Howard I Scher, Caroline C Sigman, Gary J Kelloff

**Affiliations:** 1 New Enterprise Associates, Menlo Park, CA 94025, USA; 2 Johnson &Johnson, Radnor, PA, 19087, USA; 3 CCS Associates, Mountain View, CA, 94043, USA; 4 Critical Path Institute, Tucson, AZ, 85718, USA; 5 Fox Chase Cancer Center, Philadelphia, PA, 19111, USA; 6 Center for Drug Evaluation Research, US Food & Drug Administration, Silver Spring, MA, 20903, USA; 7 University of Michigan, Ann Arbor, MI, 48109, USA; 8 Covance Genomic Lab, Covance Central Labs, Seattle, WA, 98382, USA; 9 Daiichi-Sankyo Pharma Development, Edison, NJ, 08837, USA; 10 National Cancer Institute, Bethesda, MA, 20892, USA; 11 Georgetown University, Washington, DC, 20007, USA; 12 Veridex, LLC, Raritan, NJ, 08869, USA; 13 EMD Serono, Rockland, MA, 02370, USA; 14 University Medical Center Hamburg-Eppendorf, 20246, Hamburg, Germany; 15 Foundation for the National Institutes of Health, Bethesda, MA, 20814, USA; 16 Genentech, South San Francisco, CA, 94080, USA; 17 GlaxoSmithKline, Collegeville, PA, 19426, USA; 18 Eli Lilly and Company, Indianapolis, IN, 46285, USA; 19 Memorial Sloan-Kettering Cancer Center, New York, NY, 10065, USA

**Keywords:** Circulating tumor cells, Prognostic biomarker, Predictive biomarker, Analytical validation, Clinical validation, Biomarker qualification, Oncologic drug development

## Abstract

This manuscript summarizes current thinking on the value and promise of evolving circulating tumor cell (CTC) technologies for cancer patient diagnosis, prognosis, and response to therapy, as well as accelerating oncologic drug development. Moving forward requires the application of the classic steps in biomarker development–analytical and clinical validation and clinical qualification for specific contexts of use. To that end, this review describes methods for interactive comparisons of proprietary new technologies, clinical trial designs, a clinical validation qualification strategy, and an approach for effectively carrying out this work through a public-private partnership that includes test developers, drug developers, clinical trialists, the US Food & Drug Administration (FDA) and the US National Cancer Institute (NCI).

## Introduction

The Foundation for the National Institutes of Health (FNIH) Biomarkers Consortium is a public-private biomedical research partnership that promotes the development and qualification of promising biomarkers for the prevention, early detection, diagnosis and treatment of disease. Consortium partners include patient advocates, clinical researchers from academia and industry, industry members from both therapeutic and diagnostic industries, the NCI, and the FDA. The Cancer Steering Committee (CSC) of the Biomarker Consortium focuses specifically on research activities to further improve treatment for patients with cancer through the use of biomarkers in drug development and in the field of personalized medicine. To this end, the CSC is currently sponsoring several imaging and other cancer biomarker studies.

A particular goal of the CSC is to identify new technologies with the potential to contribute to biologically informed drug development and clinical medicine. One such area is the emerging field of CTC detection and analysis. The potential implications of successful development of appropriate technologies are very large, and exciting new technologies are under development. However, many questions remain unanswered regarding the biology of CTCs, how best to enumerate and characterize them and the path to regulatory and general clinical acceptance for technology platforms currently under development.

Because of its composition and the skill sets and activities of its member organizations, the Biomarkers Consortium is uniquely positioned to help answer some of these important questions. To this end, the Consortium sponsored a workshop on qualification and validation of CTC assays. This paper summarizes not only the discussions from that meeting, but the outcome of a series of further deliberations by a CTC Working Group (CWG) convened by the CSC. The results will be the basis of a series of clinical studies to be conducted by the CSC to help answer these CTC questions, and move the field forward.

### CTCs: A background

CTCs have been defined as cancer cells of solid tumor origin found in the peripheral blood. It is generally thought that these cells detach from primary or secondary tumors of patients with advanced cancer prior to detection in the circulation. CTCs are considered very rare, with an estimated frequency of one in 100 million to one in a billion blood cells [1,2]. CTCs have been detected in the peripheral blood of patients with advanced stages of most types of solid tumor cancers, and their presence may represent the hematogenous phase of metastasis since CTCs are often not seen until tumors have achieved neovascularization [1,3]. However, CTCs have also been detected in the peripheral blood of patients with localized cancers, which may be indicative of increased risk of progression to metastatic disease [4-10], and very early during tumor development, even at a pre-malignant stage in a pancreatic cancer model [11].

Certain phenotypic characteristics have been utilized consistently to identify CTCs in the peripheral blood. CTCs were initially characterized as non-leukocytic, nucleated cells that are typically epithelial in origin, and maintain significantly larger diameters and total membrane surface areas than normal blood cells. However, the morphological features of CTCs are now known to be less clearly defined, and may vary by disease, disease stage (at the time of diagnosis), or disease state (*e.g.*, pre- *vs.* post-treatment setting). In addition, a significant number of CTCs may lose their epithelial antigens and express the phenotypic markers of epithelial-mesenchymal transition (EMT), a phenotypic change that is thought to be an important feature in the metastatic process that allows tumor cells to travel to the site of metastasis formation without being affected by conventional treatment [12-15].

It is unknown if CTCs represent more aggressive cells than those in the tumor of origin. However, phenotypic assessments to date suggest that at least some subset of CTCs may represent viable metastatic precursor cells capable of initiating a clonal metastatic lesion [12-15]. In this context, the importance of tumor-stroma interactions in generation and/or maintenance of the cancer “stem”/tumorigenic cell status (tumor plasticity) should not be underestimated. The resulting molecular and phenotypical alterations are complex and may vary by cancer (sub) type, over time, and by stage of the disease (*e.g.*, [16]). These complexities introduce additional challenges for interpreting CTC analysis results.

The analytical methods/assays used and the degree of their validation will be critical to establishing a common set of criteria describing CTCs. Progress in use of different CTC-related platforms for specific applications in the clinic (beyond CTC enumeration being monitored at multiple time points during the course of patient’s treatment) depends on establishing such criteria, keeping in mind how well the malignant features of detectable circulating cells are defined, and in the context of other molecular findings, to what extent identified CTCs could be helpful in detecting emerging resistance to treatment. This latter aspect could be critical for early modification of therapy, thus contributing to personalized health care.

A recent insight derived from CTC analyses has involved finding clusters of CTCs in the circulation of patients with advanced cancer [17-19]. It is not clear whether these aggregates are artifacts of sample processing, or if the detection of clusters has previously been limited by the technologies used for CTC isolation, most of which are purification approaches likely to disrupt clusters [20]. Early studies suggest that CTC clusters may be relatively protected from cell death and the harsh environment and shear stresses of the vascular circulation; they may also be clinically significant; particularly the number, size, or composition of the clusters [17-19,21-23]. The presence of clusters may be a better biomarker for increased metastatic potential than single CTCs [17,18,22].

### Major questions to be answered concerning CTCs

The phenotype(s) of CTCs has not been fully defined. It is currently accepted that CTCs are morphologically EpCAM+, cytokeratin (CK)+, and CD45−. However, there is evidence that these cells can also occur in patients with benign diseases of the colon [24] and other phenotypes may be present in the blood of metastatic cancer patients, including CD45+, CK + cells. It has been shown that these double-positive cells can be the result of long storage, and probably represent artifacts (which is consistent with evidence that that CK +, CD45 + cells do not have prognostic impact [25]). Further, while CTCs are thought to seed metastatic sites from a primary tumor, there is known discordance between tumor tissue biomarker expression and CTC biomarker expression, as well as heterogeneity of biomarker expression among multiple metastatic sites [26-28]. This suggests that CTCs from metastatic sites can in themselves seed new sites (*e.g.*, [29]). In addition, recent studies have shown that CTC markers may change over the course of therapy (*e.g.*, [30]).

Identification of predictive biomarkers in CTCs has the potential to become a breakthrough in cancer diagnostics and drug development. There are many sensitive and reliable tools that may be used for the characterization of CTCs, including immunocytochemistry, immunofluorescence, laser scanning, fluorescence *in situ* hybridization (FISH), and polymerase chain reaction (PCR) techniques [9,31-33]. However, statistically meaningful numbers of CTCs may need to be isolated in order to characterize the heterogeneity, determine cellular origin (primary *vs.* metastatic tumor), and evaluate CTC response (signalling, proliferation, and apoptosis) after therapeutic interventions. Through initiatives such as the NCI Alliance in Nanotechnology in Cancer, Clinical Tumor Proteomics Analysis Consortium, The Cancer Genome Atlas, and the Physical Sciences in Oncology Centers, the NCI Center for Strategic Scientific Initiatives (CSSI) is contributing to this effort by providing the community with large-scale, reproducible genomic, epigenomic, and proteomic signatures, and with advanced, robust technologies to examine such signatures at high-throughput and high-resolution.

### The biomarker development process and development of CTCs as biomarkers

Validated CTC assays and CTCs qualified as biomarkers of cancer prognosis and response to therapy through the FDA Center for Drug Evaluation (CDER) process have the potential to improve the diagnosis and care of cancer patients, one of the goals of personalized medicine. The FDA defines a validated biomarker assay as an analytical test system with well-established performance characteristics and for which there is an established scientific framework or body of evidence that elucidates the physiologic, toxicological, pharmacologic, or clinical significance of the test results [34]. For qualification, the results of biomarker measurements are expected to have met evidentiary standards within specific contexts of use linking the biomarker result with clinical measurements, and to be reliable for specific interpretations and applications in drug development and clinical and regulatory decision-making.

There are several ways by which biomarkers gain acceptance for regulatory decision making through the FDA. On a case-by-case basis, data on candidate biomarkers that are either used to make decisions in clinical trials or are being considered as a biomarker in human safety studies may be submitted to an investigational new drug application (IND), new drug application (NDA), or biologics license application (BLA) [34]. Alternatively, a test for a biomarker that will be required to support the dose selection, safety, or effectiveness of a drug, and that a sponsor will thus integrate into the drug development program, may be submitted as part of a co-development of drug and test combination (*i.e.*, as a companion diagnostic) [35].

Finally, FDA CDER has developed a Biomarker Qualification program as an outgrowth of the Critical Path Initiative to capture consensus on the context of use and supporting data for biomarkers, and to encourage the development of new biomarkers [34]. Through this program, biomarker qualification is defined as “a conclusion that within a carefully and specifically stated ‘context of use’, the biomarker has been demonstrated to reliably support a specified manner of interpretation and application in drug development”. Once qualified, biomarkers can be used in the qualified context in INDs, NDAs, and BLAs; CDER regulatory reviews in all offices and disciplines will adhere to the qualification decision. However, an *in vitro* diagnostic device, *e.g.*, a CTC test, needed to measure a qualified biomarker is required to be cleared by the FDA Center for Devices and Radiological Health (CDRH) with data supporting the analytical and clinical performance if it is to be used clinically as a diagnostic test, *i.e.*, to inform patient management.

The voluntary process of qualification is intended for biomarkers that will be used as drug development tools [34]. Since public knowledge and access are often essential to the process, collaborative groups such as the Biomarkers Consortium can be useful sources of candidate biomarkers. The process itself provides a framework for interactions between CDER (and often other centers within FDA) and sponsors, with CDER guidance towards the compilation of comprehensive evidence to support the end result of qualification. The interactions include initial evaluation, interactive consultation and advice, and final in-depth review.

An analytically validated assay for the biomarker is a prerequisite for qualification, since the stability, accuracy, and reproducibility of the measurement will be critical to demonstrating utility. Candidate CTC-based biomarkers include the number and/or molecular sub-classification of target cells, and among the assays of interest are those that measure these parameters. Criteria for assessing the analytical validity of clinical assays have been published by the Clinical and Laboratory Standards Institute (CLSI), FDA’s Office of *In Vitro* Diagnostics, and other groups [36]. Some considerations for evaluating the analytical and clinical validity of CTC assays are listed below, and reliability of data sources for determining clinical validity are listed in Table 1.

**Table 1:** Evaluation Criteria for Sources of Clinical Assay Performance Data

**Qualitative Rankings of Data Sources and Study Designs**	
Most Reliable	Collaborative studies that use large panels of well characterized samples; summary data from well-designed external proficiency testing schemes; inter-laboratory comparison programs
Reliable	Other data from proficiency testing schemes, well-designed peer-reviewed studies, and expert panel-reviewed FDA summaries
Less Reliable	Less well designed peer-reviewed studies
Least Reliable	Unpublished and/or non-peer reviewed research; clinical laboratory, or manufacturer data; studies on performance of the same basic methodology, but used to test for a different target
**Criteria for Assessing the Internal Validity of the Studies Used to Obtain Data**	
· Adequate descriptions of the assay platform and test procedures, including the reproducibility of test results, quality control measures, and comparison to a “gold standard” reference assay.	
· Samples representative of the study population, blinded testing and interpretation.	
· Data analysis including point estimates of sensitivity and specificity with 95% confidence intervals, and sample size/power calculations.	
· Studies graded as convincing, adequate, or inadequate based on their ability to provide confident estimates of analytic sensitivity and specificity using intended sample types from representative populations.	

CTC assay analytical validation considerations include the following:

· Materials at study site, *e.g.,* sample collection tubes, shipping containers

· Materials at analytic laboratory, *e.g.,* assay-specific kit (w/instructions for use, reagents, buffers, controls, *etc.*), additional equipment (centrifuge, test tubes, micropipettes, vortex mixer, *etc.*)

· Reagent storage conditions (temperature, time, *etc.*)

· Specimen collection, *e.g.,* timing of specimen collection relative to study treatments and procedures, phlebotomy procedure (patient positioning, needle size, blood draw site, sample size and number of samples, collection tube, tube inversion for clot prevention)

· Specimen handling *e.g.*, storage time and temperature, shipping time, *etc.*

· Type of analysis (enrichment/enumeration/molecular characterization of target cells)

· What is the range of specimens tested?

· Sensitivity of assay (enrichment/enumeration/molecular characterization of all cells)

· Specificity of assay (enrichment/enumeration/molecular characterization of only target cells)

· How often does the assay give a useable result?

· How similar are the results with repeated measurements?

· What are the analytical range, reproducibility, and clinical applicability of the test?

· How similar are the results obtained within the same or in multiple laboratories using the same or different technology?

· Assay-specific controls should be run each day of analysis or when a new assay lot is used to check overall performance, including instrument, reagents, and operator technique

· Assay-specific controls should be run regularly (monthly or quarterly) to show performance at the lower limit of analysis

· Assay-specific samples should be run semi-annually to document the lower limit of quantitation

· Calibration/calibration verification (at least semi-annually)

· All participating laboratories in the same study should use common controls to document continuously across laboratory performance.

· Splitting samples across laboratories prior to the study is recommended to document assay performance across laboratories.

CTC assay clinical validity considerations include the following:

· Clinical sensitivity and specificity of assay

· Are there methods to resolve clinical false positive assay results in a timely manner?

· Prevalence (how often are the target cells detectable in the study population?)

· What is the relationship between detectable target cells and the study disease?

· Positive predictive value (what is the probability that a positive assay result means that the subject has the target disease?)

· What are the genetic, environmental, or other modifiers for detection of the target cells?

A working group formed at the NCI–European Organisation for Research and Treatment of Cancer (EORTC) First International Meeting on Cancer Diagnostics convened in Nyborg, Denmark, in July 2000 created a set of criteria for high quality reporting of biomarker studies [37]. The guidelines evaluate study design, methods, and analyses; and provide suggestions for the types of data elements that should be reported. These guidelines will be useful in obtaining data for evaluating and comparing CTC assays.

During clinical validation prospective clinical studies may be designed to show evidence of the link between the biomarker being measured and the specific biologic process or clinical outcome. Sometimes, links between a candidate biomarker and a biologic process or clinical outcome can be supported by examining retrospectively collected data or specimens for which the process or outcome in question is known, but this will generally provide only exploratory information that will need prospective confirmation. Retrospective collections of samples are not expected to contain stable/viable/molecularly unaltered CTCs, due to the labile nature of live cells once removed from the body.

### Potential contexts of use for CTCs

Many potential clinical contexts of use exist for CTCs. Detection of CTCs may potentially be used to establish a diagnosis, as an alternative to invasive biopsies for early detection of metastatic tumor tissue, and for monitoring cancer patients [21,38-40]. CTCs also have potential for investigation of heterogeneity in biomarker expression between primary tumors and distant metastases, as well as among multiple metastatic sites [9,26-28,30,41]. Particularly interesting to the Biomarkers Consortium, the numbers and characteristics of CTCs may be prognostic for survival or predictive of response to cancer therapy in general or of response to a specific therapy. Moreover, changes in CTCs during the course of therapy may serve as biomarkers of treatment response or resistance.

Assays that enumerate or report the number of CTCs in a whole blood sample of a patient with advanced cancer have been shown to be prognostic for survival [42-44]. CTC enumeration via the Veridex CellSearch^TM^ System is FDA-cleared for use as an aid in monitoring patients with metastatic breast, colorectal, and prostate cancers (FDA, CDRH). The presence prior to treatment of ≥5 CTCs in a 7.5 ml sample for metastatic breast and prostate cancers, and ≥3 CTCs for colorectal cancer is associated with decreased progression-free and overall survival, and is prognostic, regardless of therapy used. Recent studies have also shown the potential utility of the CellSearch^TM^ assay for monitoring patients with melanoma, urothelial, and lung cancer [45-48].

Beyond enumeration, molecular profiling of CTCs has the potential to provide predictive information to guide the selection of therapy [49,50]. For example, although several studies have not seen this correlation [9,41,50], other studies indicate that there is concordance for human epidermal growth factor receptor 2 (HER2) gene status between primary breast cancer tumors and CTCs. This suggests that targeting patients with HER2-positive (HER2+) CTC test results with the anti-HER2 antibody trastuzumab may significantly improve clinical outcomes for HER2+ breast cancer (*e.g.*, [51]). CTC characterization may also aid in accelerating drug development and approval, since the focus of many current oncology drug discovery efforts is developing targeted therapies against signalling proteins implicated as important drivers of aggressive tumor growth and survival. A key step in the successful clinical development of such agents is the identification of responsive or resistant patient populations with predictive biomarkers for these therapies. The study of detailed molecular signatures for individual CTCs may also provide leads in new drug discovery by assessing the markers unique to progression so that relapsing patients can be treated for their current “molecular disease”.

As cancer cells can be more frequently sampled in circulation than through tumor biopsy, characterization of CTCs over time can provide an opportunity to better assess dynamic physiologic responses to treatment. Measuring CTCs sequentially during the course of therapy may reveal tumor evolution under therapeutic “natural selection” and permit the identification of biologic determinants of drug resistance or progression (*e.g.*, secondary mutations) [9,52,53]. In this regard, evolutionary biologists working with the NCI Office of Physical Sciences-Oncology, are discovering instantaneous and long-term responses in more than 150 patients by examining robust data generated by high-content CTC platforms using ~2500 samples collected at regular timed intervals.

Post-therapy changes in CTCs might be an easily accessible intermediate point-of-response efficacy. Further, a number of cases have shown that CTC numbers change prior to changes being seen with anatomical imaging, and that CTC analysis may be a more robust surrogate of survival than anatomical imaging; so CTCs potentially may have more utility in the clinical setting [9,10,54]. CTC evaluation might ultimately be used as a surrogate measurement of clinical benefit and therefore an endpoint in clinical research.

A major challenge in processing and analyzing CTCs is their low concentration in blood. For example, detailed standard protocols for controlling pre-analytic variables associated with collection and handling of blood samples are likely needed to ensure reproducibility of assay results (see below under Standards). A wide range of assay platforms is currently under development for analysis of CTCs [29,32,50,55]; the developers seek to optimize both sensitivity and specificity for detecting CTCs and to avoid generation of artifacts from extensive processing. Whole blood sample enrichment for CTCs generally involves density gradient centrifugation, immunomagnetic isolation, microfluidics, or some combination of techniques. CTC enumeration platforms are generally image-based approaches using immunocytochemistry or laser scanning cytometry, and CTC characterization techniques are typically nucleic acid or protein-based molecular assays, imaging, or a combination of molecular and imaging methods. As suggested by the background discussion above, each assay platform will likely have strengths and weaknesses in evaluating various aspects of CTCs. For instance, assays using immunomagnetics with anti-EpCAM antibodies are very efficient at enriching for epithelial cells but may miss cancer cells that have undergone EMT.

A number of promising techniques reported in the past few years use microfluidic devices to isolate CTCs from whole blood. In addition to enabling detection of CTCs in small blood samples, key design features of these techniques are flow patterns to optimize exposure of blood cells to the CTC detection method (often anti-EpCAM antibodies) and to minimize nonspecific leukocyte binding, as well as provision of platforms for molecular characterization of the captured cells. For example, among the microfluidics-based devices is one in which blood is pumped across a silicon-etched chip containing thousands of microposts fitted with anti-EpCAM antibodies. EpCAM-positive cells attach to the microposts and are then analyzed using dyes and imaging [7]. An enhanced version of this CTC Chip uses microvortices in a herringbone pattern to increase the number of interactions between CTCs and the antibody-coated chip surface [17]; DNA from cells captured on the chip are extracted and analyzed for specific molecular targets [52]. Another microfluidics device contains sinusoidal micro channels coated with anti-EpCAM antibodies to capture CTCs; the captured cells are detached from the micro channels using trypsin and passed through a conductivity sensor on the device for enumeration [56]. Specific to prostate cancer, a microfluidic device with a three-dimensional flow pattern has been described that uses anti-prostate specific membrane antigen (PSMA) antibody to capture CTCs (PSMA+, CD45− cells) [57]. Captured cells are fixed on the device and stained for PSMA, EpCAM, and presence of a nucleus; CTCs are enumerated with the aid of confocal microscopy. The captured cells can be made permeable to enable detection of intracellular antigens (*e.g.,* TMPRSS-ERG fusion protein) or lyzed for RNA extraction. Table 2 lists some of the more widely publicized CTC assays and assay platforms being developed for commercial use, including those with microfluidic and microarray technology.

**Table 2:** CTC Assays and Technologies

**Assay/Technology Name**	**Manufacturer/Developer**	**Assay Outcome**	**Target Cancer(s)**	**Technology/Process**
**AdnaTest**	AdnaGen, Langenhagen, Germany	Enrichment/Characterization	Breast, Prostate, Colon	Immunomagnetic-based EpCAM enrichment using labelled beads incubated with the whole blood sample. Unlabeled cells are removed; labelled cells are then lyzed. RNA is isolated, followed by multiplex RT-PCR (GA733-2, HER2, MUC1) to detect specific tumor biomarkers.
**Anti-EpCAM/Anti-CK Antibody CTC Enrichment**	Glenn Deng, Stanford University, Stanford, CA	Enrichment/Enumeration	Metastatic Breast Cancer	CTC enrichment assay using the combination of anti-CK and anti-EpCAM antibodies. Image analysis performed using the Ariol® system. CTC identification with brightfield and fluorescence labelled anti-CK, anti-CD45, and 4′,6-diamidino-2-phenylindole (DAPI) images.
**ApoStream**^**TM**^	ApoCell	Enrichment	Prostate, Lung	Isolation of CTCs in a whole blood sample using dielectrophoretic field flow fractionation (DFFF), which separates cells based on differing dielectric properties. See also DFFF technology entry below.
**autoMACS/MACS (Magnetic Activated Cell Sorting System)**	Mitenyi Biotec, Bergisch Gladbach, Germany	Enrichment	----------	Utilizes an immunomagnetic column to capture cells with various antigens (EpCAM, pan-CK, HER2/neu, or CD45). Manual or semi-automated system. These viable cells are available for subsequent analysis following enrichment.
**Bioflux**	Fluxion Biosciences, South San Francisco, CA	Enumeration	----------	Well Plate Microfluidic™ technology to obtain physiologically-relevant data from cell-based assays. Data acquisition obtained in brightfield, phase, fluorescence, and confocal high-resolution microscopy.
**CEER (Collaborative Enzyme Enhanced Reactive) Immunoassay**	Prometheus Laboratories Inc.	Characterization	----------	Multiplexed protein microarray platform that measures the expression and activation of specific cancer pathways with high levels of sensitivity and specificity.
	Bayer Schering Pharma AG, Germany			
**CELLection™ Epithelial Enrich**	Traci Libby, Invitrogen, Carlsbad, California	Enrichment	----------	Positive isolation. Obtain up to 5 log enrichment of viable epithelial tumor cells that are suitable for immunocytochemical staining or any other downstream application.
**CellSearch™**	Veridex	Enrichment/Enumeration	Metastatic Breast, Colon, Prostate, Lung, Melanoma, Urothelial Cancer	Automated immunomagnetic enrichment and staining system for quantification of CTCs in whole blood samples. CTCs are enriched using ferrofluids coupled to anti-EpCAM antibodies and identified by cytokeratin staining using fluorescent anti-CK antibodies, as well as counterstaining with anti-CD45 antibodies. Currently, the only diagnostic test cleared by the FDA.
**ClearCell System** (**CTChip and Clearcell Unit)**	Clearbridge Biomedic, Singapore	Enrichment	----------	Detects and isolates intact, viable CTCs from small quantities of whole, unprocessed blood. Isolated CTCs can then be stained directly on the CTChip® for identification, or retrieved for further molecular analysis.
**CTC Chip**	Dan Haber and Mehmet Toner, Dana Farber and MGH, Boston, MA	Enrichment/Enumeration	Breast, Colon, Lung, Prostate, Pancreas	Enrichment using microfluidic technology—whole blood is pumped across a silicon-etched chip that contains 78,000 microposts fitted with anti-EpCAM antibodies. EpCAM-positive cells attach to the microposts and are then detected by a camera. Includes a chamber to enclose the fluid and chip and a pneumatic pump.
	Developers:			
	On-Q-ity, Waltham, MA			
	ICx Biosystems, San Diego, CA			
	Johnson & Johnson			
**CTC Membrane Microfilter**	Richard Cote, Ram Datar, University of Miami, FL	Enumeration	Prostate	Stepwise photolithography process that produces controlled-size pores designed to exploit cell size differences between tumor and normal blood cells. Combined with quantum dot-based immunofluorescence detection for CTC characterization.
**Cytoscale CTC Assay**	Hsian-Rong Tseng, University of California, Los Angeles	Enumeration	Prostate, Breast, Colon and Kidney	Antibody cell-surface marker capture enhanced by nanostructures; immunohistochemistry staining for cell identification.
**DEPArray**	Nicolo Manares, Silicon Biosystems, SpA, Bologna, Italy	Enumeration	----------	Cell microarray for individual cell manipulation and detection. The base is a microelectronic active silicon substrate embedding control circuitry for addressing each individual dielectrophoretic (DEP) cage (cage size can be set to accommodate a single cell). The system allows detection and sorting of rare cells and sorting by morphological parameters such as shape, nucleus-to-cytoplasm ratio, fluorophores co-localization (by image-based selection).
**Dielectrophoretic Field Flow Fractionation** **(DFFF)**	Peter Gascoyne, MD Anderson Cancer Center, Houston, TX	Enumeration	----------	Cell-separation technique that exploits the differences in density and dielectric properties of cells to aid isolation of CTCs from clinical blood specimens. See also ApoStream™ assay above.
**Dylight Technology**	Medical University Graz, Austria	Enumeration	Breast	Immunofluorescence method for identifying CTCs that utilizes staining for multiple markers, including CD44, ALDH1, and CK using DyLight dyes; and subsequent analysis by novel DyLight technology.
**Dynabeads® CD45**	Traci Libby, Invitrogen, Carlsbad, California	Enrichment	----------	Dynabeads® are coated with anti-CD45 monoclonal antibody for efficient depletion of human leucocytes in whole blood samples to enrich epithelial tumor cells.
**Dynabeads® Epithelial Enriched**	Traci Libby, Invitrogen, Carlsbad, California	Enrichment	----------	Dynabeads® are coated with the monoclonal antibody BerEP4 against the human epithelial antigen, EpCAM. Enriched tumor cells are lysed for mRNA isolation and RT-PCR amplification.
**Epic HD-CTC Assay**	Epic Sciences, Inc.	Enumeration/Characterization	Prostate, Breast, Pancreas	CTC detected in peripheral blood through red blood cells lysis and fluorescentlylabeled antibodies. See also FAST Cytometer entry.
**EPISPOT (EPithelial ImmunoSPOT)**	Catherine Alix-Panabieres and Klaus Pantel, Laboratoire de Virologie, Hôpital Lapeyronie, CHU Montpellier, France & UKE, Hamburg, Germany	Characterization	Breast, Prostate, Colon	After depletion of CD45 positive cells, remaining cells in a whole blood sample are cultured for 24 hours on a membrane coated with antibodies that detect proteins shed from viable CTCs by secondary antibodies labelled with fluorochromes.
**FAST Cytometer** **(Fiberoptic Array Scanning Technology)**	Peter Kuhn, Scripps Institute, La Jolla, CA	Enumeration/Characterization	Metastatic Breast Cancer	Fluorescence cytometry combined with an automated digital microscopy imaging system. Immunofluorescently labelled CTCs are detected on a glass slide using laser-printing optics, which can scan 300,000 cells per second. See also Epic HD-CTC Assay entry.
	Robert Bruce, Scripps Palo Alto Research Center, Palo Alto, CA			
**Flow Cytometry**	Jeannie Gaylor, Becton-Dickinson, San Jose CA	Enrichment/Enumeration	----------	Multiple reagents and systems adaptable to analysis or sorting of CTCs.
**HB-CTC (Herringbone-Chip)**	Mehmet Toner and Daniel Haber, Massachusetts General Hospital (MGH) and Harvard Medical School	Enrichment/Enumeration	----------	A high-throughput microfluidic mixing device which provides an enhanced platform (over the CTC-chip) for CTC isolation where microvortices are utilized to significantly increase the number of interactions between target CTCs and the antibody-coated chip surface.
**ISET (Isolation by Size of Epithelial Tumor cells)**	Metagenex, Paris, France	Enrichment	----------	CTCs are separated from other cells in whole blood by size via vacuum filtration. This technique is gentle and produces viable cells that can be further analyzed following enrichment.
**IsoFlux™ Rare Cell Access System**	Fluxion Biosciences, South San Francisco, CA	Enrichment	----------	Proprietary microfluidic technology to isolate rare cells with high efficiency. The system incorporates CellSpot™ Technology to produce a highly concentrated sample that is optimized for downstream molecular analyses.
**Laser Scanning Cytometry**	Maintrac, Bayreuth Germany	Enumeration/Characterization	Breast, Colon, Prostate, Sarcoma	Custom laboratory analysis service performed on slides using a variety of fluorochrome-labelled antibodies or other techniques (*e.g.,* estrogen receptor, HER2, prostate-specific antigen, FISH, terminal dUTP nick end labelling). Traceable single cell detection within 1 million cells.
**Laser Scanning Cytometry (LSC)**	ApoCell	Enumeration	----------	Proprietary microscope-based immunofluorescent image analysis.
**Lymphoprep™ (Ficoll-Isopaque)**	Axis-Shield PoC, Oslo, Norway	Enrichment	----------	Separates mononuclear cells from other cells in whole blood based on cell density.
**MagSweeper**	Stephanie Jeffrey and Ronald W. Davis, Stanford University, Stanford, CA	Enrichment/Enumeration	Metastatic Breast	Automated immunomagnetic enrichment―gently enriches target cells and eliminates cells that are not bound to magnetic particles. Isolated cells can be extracted individually based on their physical characteristics to deplete any cells nonspecifically bound to beads. Processes 9 mL of blood per hour and captures >50% of circulating epithelial cells as measured in spiking experiments.
**Multiphoton Intravital Flow Cytometry**	Philip Low, Purdue University, West Lafayette, IN	Enumeration	Prostate	Noninvasively counts rare CTCs *in vivo* as they flow through the peripheral vasculature. The method involves intravenous injection of a tumor-specific fluorescent ligand followed by multiphoton fluorescence imaging of superficial blood vessels to quantitate the flowing CTCs.
**Nanodetector**	Gilupi, Potsdam, Germany	Enrichment/Enumeration	Breast, lung, prostate	The nanodetector (functionalized structured medical wire, FSMW) is inserted into the patient’s arm vein via a standard 20-gauge needle. The nanodetector consists of a medical stainless steel wire, coated with a gold layer and a hydrogel functionalized with an anti-EpCAM antibody. During the 30 min application in the vein, up to 1,500 mL of blood including the respective CTC pass the nanodetector and enable a high number of CTC to be bound by the anti-EpCAM antibody.
**Negative Enrichment OMS**	Jeffrey Chalmers, Cleveland Clinic, Cleveland, OH	Enrichment/Enumeration	Head & Neck, Breast	Red cell lysis, immunomagnetic labelling and subsequent depletion of CD45+ cells (leukocytes). Remaining cells may be further characterized (epithelial cells, cells undergoing EMT).
	PerCelleon, LLC			
	Stem Cell Technologies			
				
**Nucleopore Assay**	Whatman International Ltd., UK	Enrichment	----------	CTCs are separated from other cells in whole blood by size via vacuum filtration.
**OncoCEE-BR™**	Biocept, Inc. and Clarient, Inc.	Enumeration/Characterization	Breast	OncoCEE™ captures CTCs via a microfluidic system that uses multiple antibodies for capture followed by detection using CEE-Enhanced staining and then detects their HER2/*neu* status via FISH.
**OncoQuick**	Greiner Bio-One, Germany, North Carolina	Enrichment	Breast, Colon, Others	Centrifugal separation using optimized liquid media based on tumor cell buoyant density only. Achieves enrichment of up to 6 logs from approximately 10^4^ total mononuclear cells. Validated with spiking studies.
**Optofluidic Intracavity Spectroscopy (OFIS)**	David Kisker eOptra, Longmont, CO	Enumeration	----------	OFIS has been used to investigate the properties of several tumor cell lines and compared the results to cells from peripheral blood. The results suggest that a unique optical signature may be a characteristic of many tumor cells. This signature may offer a complementary tool to molecular methods for detection and enumeration of CTCs. In addition, by using dielectrophoresis to trap and steer cells, it is possible that induced changes in the OFIS spectrum may detect other characteristics of tumor cells, as well as transport and sort them according to those characteristic properties.
**Photoacoustic Detection**	John Viator, University of Missouri, Columbia, MO	Enumeration	Melanoma, Breast Cancer	Flowmetry system in which blood samples are irradiated with laser light, and photoacoustic waves from cancer cells are detected and counted (uses melanin in melanoma, gold-tagging of other cancer cells).
**RARE (RosetteSep-Applied Imaging Rare Event)**	StemCell Technologies, Vancouver, BC	Enrichment	----------	Negative selection technique where tetrameric antibody complexes crosslink CD45-expressing leukocytes to red blood cells in whole blood. These complexes pellet to the bottom of the tube when centrifuged due to increased density, enriching CD45-negative cells (CTCs).
**RoboSep/EasySep™**	Stem Cell Technologies, Vancouver	Enrichment	Myeloma, Epithelial Tumors, CD45 depletion	Immunomagnetic nanoparticle-cell complexes are captured in tubes and unlabeled cells are poured off. Adaptable to custom CTC antibody surface antigens. Manual or semi-automated systems.
**ScreenCell® Cyto, ScreenCell® CC, ScreenCell® MB**	ScreenCell Company Biopark 12 rue J-A de Baïf 75013, Paris	Enrichment	----------	The ScreenCell® Cyto device isolates rare tumor cells, with a high recovery rate. The ScreenCell® CC device allows isolation of either fixed or live cells. Fixed cells are well preserved morphologically. Immunocytochemistry and FISH assays can be performed directly on the filter. Isolated live cells are able to grow in culture. High-quality genetic materials (DNA, RNA) can be obtained directly from tumor cells isolated on the ScreenCell® MB device filter. The ScreenCell® devices may be able to simplify and improve noninvasive access to tumor cells due to their reduced size, versatility, and capacity to isolate CTCs within minutes.
**Single Cell Gene Expression with BioMark™ Real-Time PCR System**	Fluidigm Corporation, South San Francisco, CA	Characterization	----------	Allows high-throughput cell-line studies to determine individual cell behavior and is suited to determine single-gene cell expression levels in CTCs. Results are presented as a heat map, with individual assays on the X-axis and individual cell samples on Y-axis. The intersection of each assay and sample is an individual real-time qPCR reaction.
Supervised* Automated Microscopy	Iqbal Habib, Ariol, Genetix, Boston MA, San Jose CA	Enumeration/Characterization	Breast, Others?	Commercial component to automatically track, review, and enumerate immunocytochemically stained candidate CTCs. Nuclear, shape factor morphology image analysis system with computer display.
**TelomeScan (OBP-401)**	Oncolys BioPharma, Tokyo	Enumeration/Characterization	Gastric, Breast	Uses a virus vector for CTC detection. The virus is incubated with whole blood sample for 24 hrs and replicates with cancer cells, incorporating the GFP marker into them. CTCs are then detectable by fluorescence system analysis of cell preparation on slide. Potential for *in vivo* transfection and detection of GFP + CTCs in capillary bed of patient.
**Vita-Assay** **[Functional Collagen Adhesion Matrix (CAM)]**	Wen-Tien Chen, Vitatex, Stony Brook, NY	Enumeration	Prostate	CAM ingestion. Enables molecular characterization of captured cells.

(Commercial and Being Developed for Commercial Application).

### Selection of CTC technologies for clinical development

One of the goals of the Biomarkers Consortium Workshop was to bring together experts in the field of CTC research to identify and implement practical ways of comparing the performance of the most promising CTC assay technologies at equivalent levels of development as an initial step in clinical validation. This work, viewed as a preliminary step, was necessary to determine the value of a particular technology for inclusion in clinical trials. Discussions from the workshop and recommendations from subsequent CWG meetings are summarized below.

#### Practical application

Selected CTC assays could range from relatively low technology immunoassays for enumeration to more advanced technology platforms for molecular analysis, but priority should be given to examination of technologies with robust analytical validation and potential for providing data to address the key questions regarding biology and clinical significance of CTCs.

Clearly, different technologies may be more or less appropriate for specific applications, and more than one assay may be required for different contexts of use in a given patient. For example, a technology optimized for enumeration might not be optimal for providing enriched or purified CTC populations for detailed biological characterization. Therefore, no simple generalization can be made about the “ideal CTC technology”. Nevertheless, general comments can be made regarding characteristics which each technology should possess.

For example, the CTC technologies selected for evaluation should be ready to use in a clinical laboratory setting (see Table 3). Standard reference materials for the assay should be available and readily accessible by laboratories involved in clinical studies, and standards should be in the clinically informative range (able to show that quantitation or other analysis is accurate and reproducible at the cut-off level). As noted above, the assays selected should already have published performance data with a known performance at the level that is appropriate for use in clinical trials; depending on the technology, there should also be independent analytical validation for different organ systems. The effects of pre-analytical variables related to biospecimen acquisition, processing, and transportation should be predetermined for each assay type and serve as the evidence base for standardized procedures to obtain and handle assay samples. Each assay should demonstrate a relevant dynamic range in response; where appropriate, the assay should provide capabilities for identifying biologically relevant point mutations and ideally capture viable cells for additional analyses. Each testing laboratory needs to validate the assay using the manufacturer’s published data as guidance in developing internal validation criteria.

**Table 3:** CTC Assay Clinical Readiness Evaluation

**Assay Validation**	
**Pre-Analytic**	How is specimen collected (venous route, body position, draw order, tourniquet time, needle bore, tube type)?
	When is specimen collected (time of day, relative to treatment, relative to infusates)?
	How is specimen stored (time and temperature)?
	How is specimen handled (shipping, transfers)?
**Analytic**	Sensitivity (lower limit of quantitation)?
	Reportable range?
	Specificity?
	Reproducibility?
	Robustness?
**Post-Analytic**	How is data reported?
	How is data analyzed?
	What are the reference intervals?
**Clinical Feasibility**	
· Are there analytically valid results when tested in appropriate preclinical models?	
○ with use of clinically relevant/feasible specimen acquisition?	
○ with use of clinically relevant specimen handling procedures (both at the point of acquisition and in the receiving laboratory)? These processes should be tracked and recorded.	
○ with use of clinically relevant collection scheduling?	
**Therapeutic Relevance**	
· For predictive biomarkers, is there a relationship between dose/exposure, quantifiable target modulation, and disease outcome?	
· For prognostic biomarkers, is there a relationship between baseline levels and survival?	

#### Standards

Workshop participants and the CWG strongly endorsed the development of standard language for describing CTCs and of standard operating procedures (SOPs) for clinical evaluation of CTC assays prior to any testing on patient specimens. For example, different CTC subtypes may need to be defined, and the definitions should be context-dependent. Creating a panel of criteria for describing CTCs that together can provide statistical confidence provides an alternative approach.

Pre-analytic protocol standards for acquiring, handling, and transporting samples, and acquiring, processing, and interpreting assays are needed to allow collaboration among centers at a level of detail sufficient to encompass all likely variables. These standards should enable comparisons across platforms, contexts of use, and time. Efforts should be made to develop protocols for evaluating technologies that are uniform and clinically meaningful with regard to well-defined parameters for specific applications. Though ideally flexible enough so that new technologies can be easily integrated, the standards should be evaluated rigorously.

While specific procedures for sample acquisition, handling, and processing should probably be dictated by the analysis to be done, general recommendations for sample handling should be made. Pre-analytical variables, including fasting status, time of day, body positioning during the blood draw, and venipuncture needle size should be standardized. It also may be desirable for CTC assay platform companies to supply completely self-contained collection kits to minimize the pre-analytic variability. Procedures for handling acquired samples, including sample transport and storage, should also be standardized and deviations tracked variables related to sample collection and analysis are listed following:

· Sample collection kit with instructions for use

· Shipping container

· Temperature monitoring strips

· Appropriate analytical instrumentation

· Analytical reagents

· Standards

· Multiple levels of controls

· Reagent storage temperature

· Reagent storage time

· Patient preparation—for example, fasting status

· Timing of sample collection in study protocol

· Sample collection method

· Number of specimen collected, including duplicates

· Subject positioning during sample collection

· Specimen transport time

· Specimen storage temperature

· Specimen storage time

· Assay-specific SOPs

· Lot-specific control testing

· Levels of quality control testing (daily, monthly)

· Lab certification

· Assay platform training

All testing should use rigorous criteria such as Clinical Laboratory Improvement Amendments (CLIA) or good laboratory practice (GLP) standards. Early-stage development studies of clinical assays are not subject to CLIA regulation; however, clinical trials where biomarker assays are integral to the design need to be done in CLIA-certified laboratories, and if the results of the assay are used to make treatment decisions, FDA review is generally needed. Biomarker assays are considered to play an integral role when done in real time and the results used for trial eligibility or to make individual patient decisions—for example, to stratify patients, assign a patient to a specific treatment arm, or decide whether to escalate dose or stop treatment [58].

The fragility/lability of tumor cells introduces an important source of variability in the evaluation of CTC assay platforms that analyze captured whole cells, since CTC apoptosis begins very early after separation from the tumor of origin and after removal of blood from the patient [59-61]. Incorporating cellular preservatives in the collection of peripheral blood samples has been shown to stabilize CTCs for up to 96 hours [25,32,61]. Standard use of this procedure would allow greater flexibility in sample storage and shipping, which in turn may allow the inclusion of more enumeration assay technologies in comparison evaluations, and make their utilization in the clinic more practical.

Archived CTC samples may be adequate for molecular-based assays, assuming that one is able to demonstrate that the cells of interest have survived the archiving procedure and duration in numbers and biological condition that represent their state when collected. However, access to systematically collected and appropriately well-preserved patient samples with linked clinical outcome data is essential [62]. For CTC studies in general, archived tumor samples may be difficult to obtain, suboptimal for certain molecular assays, or not representative of a patient’s disease at the time of treatment. The NCI has provided detailed recommendations related to biospecimen and data quality in the 2010 Revised NCI Best Practices. The NCI Office of Biorepositories and Biospecimen Research (OBBR) is one resource for well-defined archived samples that are collected using the most stringent SOPs.

Established in 2005, a goal of OBBR is to develop a common biorepository infrastructure to facilitate multi-institutional genomic and proteomic studies. Partnering with an organization like OBBR to develop CTC-specific SOPs, as well as a source of clinical samples for assay comparison studies, should be considered.

### Initial interassay comparison on clinical samples

For most new CTC technologies reviewed, it was noted that too little systematically collected data were available to allow evaluation of potential clinical utility or to compare with other assays. Beyond analytical validity, foundational data on the individual assays are needed, such as frequency of detection in specific patient populations and consistency of the results in a given patient in determinations on multiple days and at different time intervals, as well as the capability for detecting quantifiable changes to a patient’s results after appropriate treatment.

Specificity of a CTC assay depends on a particular test result representing the target tumor cells, as opposed to normal hematopoietic or circulating epithelial cells. This distinction can often be made by either visual inspection by a trained cytopathologist or by determination of molecular markers, or a combination of these techniques. For example, Meng *et al.* have demonstrated that in patients with metastatic breast cancer, epithelial cells identified using an immunomagnetic separation system with anti-EpCAM as the capture antibody are highly aneusomic and almost certainly malignant and not normal [60]. Further, Shaffer *et al.* demonstrated that cells captured by CellSearch™ expressed prostate specific antigens and had molecular features of malignancy as determined by FISH analysis [38].

However, it is conceivable that not all cells that meet visual or molecular criteria for cancer have true malignant potential or, conversely, that not all cells lacking the defined criteria are nonmalignant (*e.g.*, [24,63]). Current thought is in part based on stem cell theory, which posits that only a fraction of any tumor mass, and therefore of any CTC population, is able to establish and maintain a metastasis. Also as suggested above, it is known that many cells identified in the circulation are already undergoing, or have completed, apoptosis. Therefore, biologic relevance is not just the presence of unequivocally malignant-appearing cells, but the biological and/or clinical significance of these cells. The latter can only be determined by correlating the presence of assay metrics (presumed CTCs) with some clinically or biologically important outcome, such as tumor regression, subsequent recurrence, new primary cancer, or death.

The implication of biologic significance is that one cannot conclude that a new assay is superior to a gold-standard assay by mere comparison of assay metrics/unit blood between the two. One must determine whether a presumably more sensitive assay retains, or exceeds, the robust separation of outcomes between patients who are positive *vs.* those who are negative for the assay, and this can only be demonstrated in well-designed clinical studies.

Direct comparison of different technologies on the same clinical samples and/or in tumor cell spiking experiments would be informative. One or two specific CTC-derived biomarkers (*e.g.*, EpCAM) could be used to identify the optimal assay technologies. Gold-standard technologies are needed for comparisons, based on whether the evaluation is quantitative (enumeration of CTCs) or qualitative (molecular characterization of CTCs). CellSearch™ could serve as an anchor in the short term for assessments of newer enumeration assay technologies, building on prior clinical evidence for its predictive and prognostic capability.

The CWG discussed approaches to carry out this evaluation in which the Biomarkers Consortium would provide a framework wherein laboratories studying the selected technologies would have access to matched pre- and post-treatment clinical samples from multiple clinical settings as obtained by the OBBR or an equivalent source. The samples could be from patients treated with approved therapies (*e.g.*, the control arms of randomized clinical trials), well-annotated regarding patient characteristics and outcomes, and collected and processed according to standard protocols as described above. Fresh blood samples may be needed for these studies since archived samples may contain degraded cells and no longer reflect the disease state of the donor at the time of collection. The samples could be analyzed by multiple methods, would be blinded to the analyzing laboratory, and results data would be compiled by the CWG prior to release back to the assay developer. This approach would yield information regarding whether different technologies are detecting different CTC populations and provide information for definitions of analytically valid biomarkers that may need to be established for these different populations.

### Clinical trial design considerations

Despite the fact that research aimed at defining and understanding the biology of CTCs is still ongoing and many promising assays are still early in development, the Workshop participants and CWG believe that it is crucial to establish clinical validation and qualification trials soon with the goal of determining the true clinical relevance of CTCs and specific assays of CTCs. In this regard, the excellent on-going qualification work in collaboration with FDA regarding the use of CTCs as an efficacy response surrogate biomarker for survival in patients with advanced prostate cancer [38,43] was acknowledged.

Beyond clinical validation of specific assays, CTC qualification studies should initially seek to answer clinically relevant questions, such as a) to determine the potential utility of CTCs in early clinical development of drugs (phase 1 and 2 trials); b) to determine if enumeration could be an early efficacy response marker; c) to help drive the use of enumeration as a surrogate marker of response to therapy; d) to investigate whether CTC evaluations could augment or replace imaging for response monitoring; and e) to investigate whether the molecular analysis of CTCs could replace tumor biopsies for patient stratification for targeted therapies. In the short term, there is general interest in the application of CTC enumeration for determining response to therapy; therefore, association of CTC measurements with complete response, partial response, stable disease, and progressive disease would be appropriate endpoints for initial clinical trials.

Several trial designs may be useful in addressing these questions regarding evaluation of clinical validity of CTC technologies and qualification of CTC measurements as biomarkers of response to therapy. First, CTC substudies may be added to ongoing trials where the study treatment choices and primary endpoints (early endpoints such as objective response, as well as clinical outcomes) would be dictated by the parent trial. Single-arm studies could be used to explore changes pre- and post-therapy and correlation with outcomes, thus assisting with determining cut points. As single-arm trials can only evaluate prognostic potential, two-arm, two-drug substudies could also be used to compare results with different therapies to evaluate the predictive potential of CTCs. Alternatively, the CTC study may be fully integrated into the trial design. In this design, patients would be randomized on the basis of the presence or absence of CTCs. The FDA has recommended this study design to evaluate diagnostic tests for use in the selection of drug therapy.

**Figure 1:**
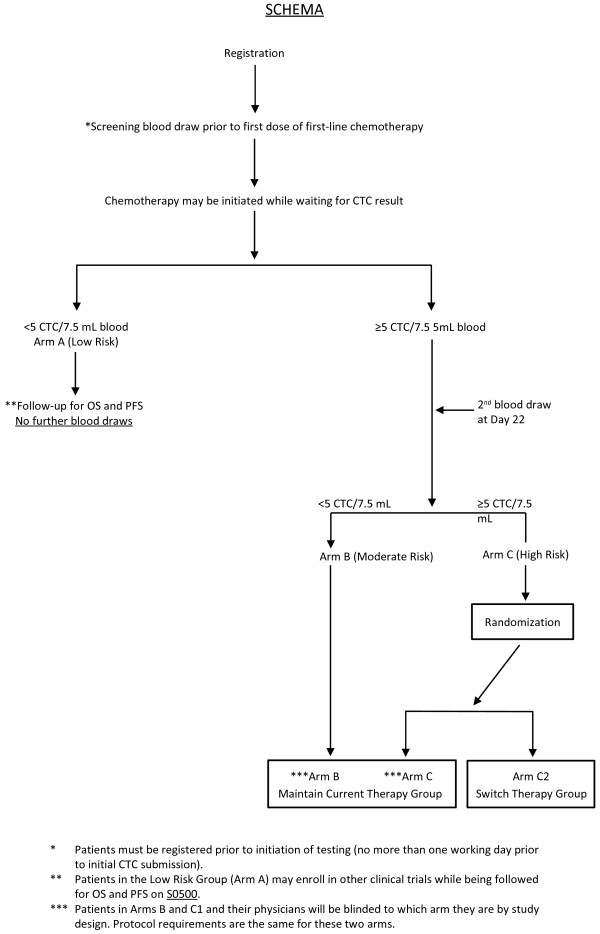
**Schema of Southwest Oncology Group (SWOG) Study S0500.** This clinical study is evaluating the use of CTC levels in managing the treatment of metastatic breast cancer patients. Baseline CTC levels are determined using the CellSearch System™. Patients with CTC levels <5 CTCs/7.5 ml receive no further therapy, but are followed for progression-free survival (PFS) and overall survival (OS) (Arm A). For the remaining patients (≥ 5 CTCs/7.5 ml blood), CTCs are measured at specified time points during the course of chemotherapy. Patients with <5 CTCs/7.5 ml blood at 22 weeks continue with their current chemotherapy (Arm B). Patients with ≥5 CTCs/7.5 ml blood at this time point are randomized to current therapy (Arm C1) or a different therapy (Arm C2). Patients are followed for PFS and OS. (Reprinted by permission from the American Association for Cancer Research: Hayes DF, Smerage J: Is there a role for circulating tumor cells in the management of breast cancer? Clin Cancer Res 2008, 14 (12):3646–3650 DOI:http://dx.doi.org/10.1158/1078-0432.CCR-07-4481. See reference [64]).

One example of an ongoing clinical trial to test the clinical utility of CTCs is SWOG S0500. In this trial, women starting a first line chemotherapeutic regimen for hormone-refractory metastatic breast cancer and who have persistently elevated CTCs after one cycle are randomly assigned to continue their therapy until classic evidence of progression (history, physical examination, imaging) or to change immediately to a different regimen. The goal of this study is to demonstrate an improved overall survival for women who are switched, based on a CTC test result (see Figure 1) [64], from an apparently ineffective treatment strategy to an alternative one. Patient populations where treatments have the potential to change based on the results of the trial should be targeted. The cohorts should include patients with measureable, advanced, non-hematologic cancers, as currently the most interesting and appropriate clinical application of CTC analysis is to establish its predictive value for response to therapy.

In terms of study indication, there should be no bias for target tumors other than the robustness of the available data. Robust CTC data have been obtained with the CellSearch™ System for breast, colorectal, and prostate disease. With the same technology, low numbers of CTCs are captured in the metastatic settings for colon and lung cancer; therefore, newer technologies with higher sensitivity and good specificity rates may be useful in these settings. In early disease, such as neoadjuvant settings, where fewer CTCs are expected [8,9,65], a potential benefit of more sensitive assays could be a shorter timeframe to analysis of study endpoints like the extent of residual disease after surgery as an early marker of outcome, which provides value for new drug development. In addition to more efficient enumeration, newer technologies provide the promise of molecular profiling of tumor response to therapy in all the clinical designs described.

### A clinical validation and qualification study strategy and process

The CSC of the Biomarkers Consortium is interested in supporting a series of studies to help resolve important pre-competitive issues around CTCs, with the overall objectives of providing evidence toward qualification of CTC derived biomarkers for assessing prognosis pre-therapy, establishing CTC number as a surrogate for survival, and determining whether the molecular profile of CTCs predict response to treatment.

The initial goal of the proposed strategy will be to demonstrate the robustness of candidate CTC assay technologies currently under development, prior to clinical studies, in order to determine which of the platforms are closest to being ready for validation in the clinical trial setting. The proposed studies will evaluate newer CTC enumeration assays considered to be the most promising technologies using criteria created by the CSC and CWG against the FDA-cleared analytically valid CellSearch™ assay. The most promising CTC molecular characterization assays will also be compared using these criteria. Additionally, recognizing the need for standardizing procedures for specimen collection and processing to ensure the reliability of CTC measurements, the CWG will develop these procedures for CTC assays evaluated in this project. A small pilot in this regard was recently presented at an OBBR symposium, where CSSI, Offices of Physical Sciences-Oncology (OPSO), and OBBR are collaborating to examine the potential of using high-content systems to measure the effect of pre-analytical variables on biophysical parameters associated with biospecimens.

For cell spiking experiments, cell lines considered appropriate for the selected technologies will be obtained and cultured in standard conditions. Fluorescent labelling of target cells will be performed using standard cell labelling kits. Multiple samples from a given cell suspension will be counted and averaged to produce a mean cell count with a small standard deviation in order to minimize counting errors. The cell suspensions will then be appropriately diluted and processed using the selected CTC assay technologies. Capture efficiency percentages will be calculated using criteria as defined by the CWG.

In clinical studies, triplicate specimens will be collected from subjects participating in ongoing studies with relevant indications and study populations in order to compare the candidate assays against each other. For the CTC enumeration studies, one of each set of three samples will be analyzed using the CellSearch™ assay. Similarly, for the CTC molecular characterization studies, one assay will be selected to be used as the anchor. All clinical samples will be procured through the OBBR, or using SOPs compliant with OBBR requirements.

In the clinical validation studies, CTC substudies will be conducted in prospective phase 2 or 3 clinical trials (or if the assays selected do not require fresh blood, archived samples will be accessed from completed prospective studies for retrospective analysis) in an attempt to correlate CTC measurements with clinical outcomes related to treatment response, and time-related outcomes such as time to progression or disease-free survival. Here, duplicate specimens will be collected. The CTC assays will be performed pre-therapy and at defined intervals post-therapy on anonymized patient samples collected in the trial, blinding technicians to any patient identifiers. The specimens will be collected at participating sites and forwarded to the specified clinical laboratories for processing. In addition to SOPs for variables associated with sample collection as outlined above standard protocols are needed to allow comparison of CTC assay results across technology platforms. Just as challenging is the development of standards for collecting certain data points to analyze after advances have occurred in the field, so comparisons could be made over time. The standards would have to be flexible to easily incorporate new technologies.

After performing clinical validation studies, the next stage, qualification, would be follow-on clinical studies to determine the context-of-use of specific CTC measures. These studies will most likely be conducted in the phase 3 metastatic disease trial setting for specified indications in specified study populations. Baseline CTCs would be assessed, and then additional assessments would be made at some point after the start of treatment. These assessments may be used to determine the role of CTC analysis for monitoring disease recurrence, evaluating new treatments against standard therapy, or investigating concordance between imaging and CTC analysis. If a series of studies with clinical benefit outcomes shows good correlation and reproducibility between a given CTC measure and survival, that biomarker could be considered a candidate for FDA qualification.

## Conclusions

Without question, the Veridex CellSearch™ assay and the advanced technologies now being applied to CTC detection and analysis have high promise for providing biomarkers and biomarker assays useful in oncological drug development, monitoring the course of disease in cancer patients, and in understanding the biology of cancer progression. However, many questions remain unanswered regarding the biology of CTCs, best methods for their enumeration and characterization, and the path to regulatory and general clinical acceptance for technologies currently under development. Standard protocols for acquisition and processing of blood samples, as well as sources of well-annotated clinical samples (including clinical outcomes) and application of criteria for analytical and clinical validation of CTC assays and qualification of CTC-based biomarkers are mandatory for the next steps in evaluation of these technologies. Developing and applying these standards is difficult, if not impossible, for individual research institutions and companies; this effort requires coordinated clinical trial resources for obtaining samples and infrastructure for developing standards, managing the collection and distribution of samples, and evaluation of test results, as well as input from engaged scientists and the FDA. To this end, the Biomarkers Consortium, as a public-private partnership, is proposing and planning to implement the framework described in this review.

## Abbreviations

BLA: Biologics license application; CDER: Center for Drug Evaluation and Research; CDRH: Center for Devices and Radiological Health; CK: Cytokeratin; CLIA: Clinical laboratory improvement amendments; CLSI: Clinical and Laboratory Standards Institute; CSC: Cancer Steering Committee; CSSI: NCI Center for Strategic Scientific Initiatives; CTC: Circulating tumor cell; CWG: CTC working group; DAPI: 4′,6-diamidino-2-phenylindole; DEP: Dielectrophoretic; DFFF, Dielectrophoretic field flow fractionation; EMT: Epithelial-mesenchymal transition; EORTC: European Organisation for Research and Treatment of Cancer; FDA: US Food & Drug Administration; FISH: Fluorescence in situ hybridization; FNIH, Foundation for the National Institutes of Health; FSMW: Functionalized structured medical wire; GLP: Good laboratory practice; HER2: Human epidermal growth factor receptor 2; IND: Investigational new drug application; NCI: US National Cancer Institute; NDA, New drug application; OBBR, NCI Office of Biorepositories and Biospecimen Research; OPSO, NCI Office of Physical Sciences-Oncology; PCR, Polymerase chain reaction; PSMA: Prostate specific membrane antigen; RT-PCR: Reverse transcriptase-polymerase chain reaction; SOPs: Standard operating procedures.

## Competing interests

Several of the authors are past or current employees of, stockholders in, consultants to, or have received research funding or honoraria from Veridex LLC, the manufacturer of the CellSearch System™ for detection of CTCs, or its parent company Johnson & Johnson (ND, DH, MCL, RM, KP, HS). DH has also been a consultant to Biomarker Strategies of Baltimore, Maryland on CTCs and is named as an inventor or co-inventor on patents on applying CTCs in clinical settings. MC is a consultant to Alere, Inc. on CTCs; GK is an employee and stockholder of Covance, Inc., which includes CTC assays (particularly the Veridex CellSearch™ system) among its commercial offerings; and DP has been an employee of Nodality, Inc. which is developing technology that has potential application in CTC analysis. The remaining authors have no relevant competing interests (CC, BGP, AD, GJK, PK, JL, SM, LN, SPW, EP, LR, AS, CCS).

## Authors’ contributions

All authors contributed to the scientific and technical material presented in the manuscript, either by providing information based on direct experience with CTC technologies or relevant experience in developing biomarkers for clinical use. DP, co-chair of the Biomarkers Consortium CSC and of the CWG, led overall development of the manuscript. GJK, co-chair of the Biomarkers Consortium CSC, ensured that aspects of CTC science relevant to development of CTCs as biomarkers for predictive and prognostic applications in oncologic development were incorporated in the manuscript. BGP and CCS were responsible for background research and drafting the manuscript. Note that the contributions of AD to this manuscript are from his perspective and do not necessarily represent the views of the FDA. All authors read and approved the final manuscript.

## Authors’ information

As noted above, each author contributed direct experience in the development of drugs or biomarkers, development and application of CTC technologies, or both.

## Author details

^1^New Enterprise Associates, Menlo Park, CA 94025, USA. ^2^Johnson & Johnson, Radnor, PA 19087, USA. ^3^CCS Associates, Mountain View, CA 94043, USA.^4^Critical Path Institute, Tucson, AZ 85718, USA. ^5^Fox Chase Cancer Center, Philadelphia, PA 19111, USA. ^6^Center for Drug Evaluation Research, US Food & Drug Administration, Silver Spring, MA 20903, USA. ^7^University of Michigan, Ann Arbor, MI 48109, USA. ^8^Covance Genomic Lab, Covance Central Labs, Seattle, WA 98382, USA. ^9^Daiichi-Sankyo Pharma Development, Edison, NJ 08837, USA. ^10^National Cancer Institute, Bethesda, MA 20892, USA.^11^Georgetown University, Washington, DC 20007, USA. ^12^Veridex, LLC, Raritan, NJ 08869, USA. ^13^EMD Serono, Rockland, MA 02370, USA. ^14^University Medical Center Hamburg-Eppendorf, 20246, Hamburg, Germany.^15^Foundation for the National Institutes of Health, Bethesda, MA 20814, USA.^16^Genentech, South San Francisco, CA 94080, USA. ^17^GlaxoSmithKline, Collegeville, PA 19426, USA. ^18^Eli Lilly and Company, Indianapolis, IN 46285, USA. ^19^Memorial Sloan-Kettering Cancer Center, New York, NY 10065, USA.

## References

[B1] Yu M Stott S Toner M Maheswaran S Haber DA Circulating tumor cells: approaches to isolation and characterization J Cell Biol 2011 192 373 382 10.1083/jcb.201010021 21300848 PMC3101098

[B2] Nelson NJ Circulating tumor cells: will they be clinically useful? J Natl Cancer Inst 2010 102 146 148 10.1093/jnci/djq016 20107163

[B3] Hou JM Krebs M Ward T Morris K Sloane R Blackhall F Dive C Circulating tumor cells, enumeration and beyond Cancers 2010 2 1236 1250 10.3390/cancers2021236 PMC3835128 24281115

[B4] Nagrath S Stott SL Lee RJ Yu M Ulkus LL Iafrate JA Smith MR Tompkins RG Sequist LV Haber DA Maheswaran S Toner M Detection and characterization of circulating tumor cells in localized and metastatic prostate cancer patients using CTC-Chip microfluidic technology Proc Am Assoc Cancer Res 2010 101st abstr 1136

[B5] Bertolin M Pigozzo J Koussis H Ghiotto C Valente S Michieletto S Magro C Rossi E Zamarchi R Bozza F Jirillo A Chiarion-Sileni V Amadori A Circulating tumor cells detection and evaluation of their apoptotic status in patients with localized breast cancer before and after surgery J Clin Oncol 2011 29 suppl abstr e21127

[B6] Muller V Stahmann N Riethdorf S Rau T Zabel T Goetz A Janicke F Pantel K Circulating tumor cells in breast cancer: correlation to bone marrow micrometastases, heterogeneous response to systemic therapy and low proliferative activity Clin Cancer Res 2005 11 3678 3685 10.1158/1078-0432.CCR-04-2469 15897564

[B7] Nagrath S Sequist LV Maheswaran S Bell DW Irimia D Ulkus L Smith MR Kwak EL Digumarthy S Muzikansky A Ryan P Balis UJ Tompkins RG Haber DA Toner M Isolation of rare circulating tumour cells in cancer patients by microchip technology Nature 2007 450 1235 1239 10.1038/nature06385 18097410 PMC3090667

[B8] Pierga JY Bidard FC Mathiot C Brain E Delaloge S Giachetti S de Cremoux P Salmon R Vincent-Salomon A Marty M Circulating tumor cell detection predicts early metastatic relapse after neoadjuvant chemotherapy in large operable and locally advanced breast cancer in a phase II randomized trial Clin Cancer Res 2008 14 7004 7010 10.1158/1078-0432.CCR-08-0030 18980996

[B9] Riethdorf S Muller V Zhang L Rau T Loibl S Komor M Roller M Huober J Fehm T Schrader I Hilfrich J Holms F Tesch H Eidtmann H Untch M von Minckwitz G Pantel K Detection and HER2 expression of circulating tumor cells: prospective monitoring in breast cancer patients treated in the neoadjuvant GeparQuattro trial Clin Cancer Res 2010 16 2634 2645 10.1158/1078-0432.CCR-09-2042 20406831

[B10] Xenidis N Ignatiadis M Apostolaki S Perraki M Kalbakis K Agelaki S Stathopoulos EN Chlouverakis G Lianidou E Kakolyris S Georgoulias V Mavroudis D Cytokeratin-19 mRNA-positive circulating tumor cells after adjuvant chemotherapy in patients with early breast cancer J Clin Oncol 2009 27 2177 2184 10.1200/JCO.2008.18.0497 19332733

[B11] Rhim AD Mirek ET Aiello NM Maitra A Bailey JM McAllister F Reichert M Beatty GL Rustgi AK Vonderheide RH Leach SD Stanger BZ EMT and dissemination precede pancreatic tumor formation Cell 2012 148 349 361 10.1016/j.cell.2011.11.025 22265420 PMC3266542

[B12] Aktas B Tewes M Fehm T Hauch S Kimmig R Kasimir-Bauer S Stem cell and epithelial-mesenchymal transition markers are frequently overexpressed in circulating tumor cells of metastatic breast cancer patients Breast Cancer Res 2009 11 R46 10.1186/bcr2333 19589136 PMC2750105

[B13] Polyak K Weinberg RA Transitions between epithelial and mesenchymal states: acquisition of malignant and stem cell traits Nat Rev Cancer 2009 9 265 273 10.1038/nrc2620 19262571

[B14] Bednarz N Eltze E Semjonow A Rink M Andreas A Mulder L Hannemann J Fisch M Pantel K Weier HU Bielawski KP Brandt B BRCA1 loss preexisting in small subpopulations of prostate cancer is associated with advanced disease and metastatic spread to lymph nodes and peripheral blood Clin Cancer Res 2010 16 3340 3348 10.1158/1078-0432.CCR-10-0150 20592016 PMC3042432

[B15] Joosse SA Hannemann J Spotter J Bauche A Andreas A Muller V Pantel K Changes in keratin expression during metastatic progression of breast cancer: impact on the detection of circulating tumor cells Clin Cancer Res 2012 18 993 1003 10.1158/1078-0432.CCR-11-2100 22228641

[B16] Lilly B Kennard S Differential gene expression in a coculture model of angiogenesis reveals modulation of select pathways and a role for Notch signaling Physiol Genomics 2009 36 69 78 18984672 10.1152/physiolgenomics.90318.2008 PMC2636923

[B17] Stott SL Hsu CH Tsukrov DI Yu M Miyamoto DT Waltman BA Rothenberg SM Shah AM Smas ME Korir GK Floyd FP Gilman AJ Lord JB Winokur D Springer S Irimia D Nagrath S Sequist LV Lee RJ Isselbacher KJ Maheswaran S Haber DA Toner M: Isolation of circulating tumor cells using a microvortex-generating herringbone-chip Proc Natl Acad Sci U S A 2010 107 18392 18397 10.1073/pnas.1012539107 20930119 PMC2972993

[B18] Hou JM Krebs M Ward T Sloane R Priest L Hughes A Clack G Ranson M Blackhall F Dive C Circulating tumor cells as a window on metastasis biology in lung cancer Am J Pathol 2011 178 989 996 10.1016/j.ajpath.2010.12.003 21356352 PMC3069884

[B19] Hou JM Krebs MG Lancashire L Sloane R Backen A Swain RK Priest LJ Greystoke A Zhou C Morris K Ward T Blackhall FH Dive C Clinical significance and molecular characteristics of circulating tumor cells and circulating tumor microemboli in patients with small-cell lung cancer J Clin Oncol 2012 30 525 532 10.1200/JCO.2010.33.3716 22253462

[B20] Molnar B Ladanyi A Tanko L Sreter L Tulassay Z Circulating tumor cell clusters in the peripheral blood of colorectal cancer patients Clin Cancer Res 2001 7 4080 4085 11751505

[B21] Serrano Fernadez MJ Alvarez Merino JC Martinez Zubiaurre I Fernandez Garcia A Sanchez Rovira P Lorente Acosta JA Clinical relevance associated to the analysis of circulating tumor cells in patients with solid tumors Clin Transl Oncol 2009 11 668 659 10.1007/s12094-009-0421-z 19828408

[B22] Wang ZP Eisenberger MA Carducci MA Partin AW Scher HI Ts’o PO Identification and characterization of circulating prostate carcinoma cells Cancer 2000 88 2787 2795 10.1002/1097-0142(20000615)88:12<2787::AID-CNCR18>3.0.CO;2-2 10870062

[B23] Glinsky VV Glinsky GV Glinskii OV Huxley VH Turk JR Mossine VV Deutscher SL Pienta KJ Quinn TP Intravascular metastatic cancer cell homotypic aggregation at the sites of primary attachment to the endothelium Cancer Res 2003 63 3805 3811 12839977

[B24] Pantel K Deneve E Nocca D Coffy A Vendrell JP Maudelonde T Riethdorf S Alix-Panabieres C Circulating epithelial cells in patients with benign colon diseases Clin Chem 2012 58 936 940 10.1373/clinchem.2011.175570 22205690

[B25] Riethdorf S Fritsche H Muller V Rau T Schindlbeck C Rack B Janni W Coith C Beck K Janicke F Jackson S Gornet T Cristofanilli M Pantel K Detection of circulating tumor cells in peripheral blood of patients with metastatic breast cancer: a validation study of the Cell Search system Clin Cancer Res 2007 13 920 928 10.1158/1078-0432.CCR-06-1695 17289886

[B26] Meng S Tripathy D Shete S Ashfaq R Haley B Perkins S Beitsch P Khan A Euhus D Osborne C Frenkel E Hoover S Leitch M Clifford E Vitetta E Morrison L Herlyn D Terstappen LW Fleming T Fehm T Tucker T Lane N Wang J Uhr J HER-2 gene amplification can be acquired as breast cancer progresses Proc Natl Acad Sci U S A 2004 101 9393 9398 10.1073/pnas.0402993101 15194824 PMC438987

[B27] Wulfing P Borchard J Buerger H Heidl S Zanker KS Kiesel L Brandt B HER2-positive circulating tumor cells indicate poor clinical outcome in stage I to III breast cancer patients Clin Cancer Res 2006 12 1715 1720 10.1158/1078-0432.CCR-05-2087 16551854

[B28] Chang HJ Han SW Oh DY Im SA Jeon YK Park IA Han W Noh DY Bang YJ Kim TY Discordant human epidermal growth factor receptor 2 and hormone receptor status in primary and metastatic breast cancer and response to trastuzumab Jpn J Clin Oncol 2011 41 593 599 10.1093/jjco/hyr020 21406492

[B29] Alix-Panabieres C Schwarzenbach H Pantel K Circulating tumor cells and circulating tumor DNA Annu Rev Med 2012 63 199 215 10.1146/annurev-med-062310-094219 22053740

[B30] Wang LH Pfister TD Parchment RE Kummar S Rubinstein L Evrard YA Gutierrez ME Murgo AJ Tomaszewski JE Doroshow JH Kinders RJ Monitoring drug-induced gammaH2AX as a pharmacodynamic biomarker in individual circulating tumor cells Clin Cancer Res 2010 16 1073 1084 10.1158/1078-0432.CCR-09-2799 20103672 PMC2818670

[B31] Maheswaran S Haber DA Circulating tumor cells: a window into cancer biology and metastasis Curr Opin Genet Dev 2010 20 96 99 10.1016/j.gde.2009.12.002 20071161 PMC2846729

[B32] Lianidou ES Markou A Circulating tumor cells in breast cancer: detection systems, molecular characterization, and future challenges Clin Chem 2011 57 1242 1255 10.1373/clinchem.2011.165068 21784769

[B33] Ignatiadis M Rothe F Chaboteaux C Durbecq V Rouas G Criscitiello C Metallo J Kheddoumi N Singhal SK Michiels S Veys I Rossari J Larsimont D Carly B Pestrin M Bessi S Buxant F Liebens F Piccart M Sotiriou C HER2-positive circulating tumor cells in breast cancer PLoS One 2011 6 e15624 10.1371/journal.pone.0015624 21264346 PMC3018524

[B34] US Food and Drug Administration Guidance for Industry: Qualification Process for Drug Development Tools. Draft Guidance 2010 http://www.fda.gov/downloads/Drugs/GuidanceComplianceRegulatoryInformation/Guidances/UCM230597.pdf

[B35] US Food and Drug Administration Guidance for Industry: Pharmacogenomic Data Submissions 2005

[B36] US Food and Drug Administration Draft Guidance for Industry and Food and Drug Administration Staff: In Vitro Companion Diagnostic Devices Draft Guidance 2011 http://www.fda.gov/downloads/MedicalDevices/DeviceRegulationandGuidance/GuidanceDocuments/UCM262327.pdf

[B37] McShane LM Altman DG Sauerbrei W Taube SE Gion M Clark GM Reporting recommendations for tumor marker prognostic studies (REMARK) J Natl Cancer Inst 2005 97 1180 1184 10.1093/jnci/dji237 16106022

[B38] Shaffer DR Leversha MA Danila DC Lin O Gonzalez-Espinoza R Gu B Anand A Smith K Maslak P Doyle GV Terstappen LW Lilja H Heller G Fleisher M Scher HI Circulating tumor cell analysis in patients with progressive castration-resistant prostate cancer Clin Cancer Res 2007 13 2023 2029 10.1158/1078-0432.CCR-06-2701 17404082

[B39] Serrano MJ Sanchez-Rovira P Delgado-Rodriguez M Gaforio JJ Detection of circulating tumor cells in the context of treatment: prognostic value in breast cancer Cancer Biol Ther 2009 8 671 675 10.4161/cbt.8.8.7834 19242121

[B40] Danila DC Anand A Sung CC Heller G Leversha MA Cao L Lilja H Molina A Sawyers CL Fleisher M Scher HI TMPRSS2-ERG status in circulating tumor cells as a predictive biomarker of sensitivity in castration-resistant prostate cancer patients treated with abiraterone acetate Eur Urol 2011 60 897 904 10.1016/j.eururo.2011.07.011 21802835 PMC3185163

[B41] Fehm T Muller V Aktas B Janni W Schneeweiss A Stickeler E Lattrich C Lohberg CR Solomayer E Rack B Riethdorf S Klein C Schindlbeck C Brocker K Kasimir-Bauer S Wallwiener D Pantel K HER2 status of circulating tumor cells in patients with metastatic breast cancer: a prospective, multicenter trial Breast Cancer Res Treat 2010 124 403 412 10.1007/s10549-010-1163-x 20859679

[B42] Cristofanilli M Budd GT Ellis MJ Stopeck A Matera J Miller MC Reuben JM Doyle GV Allard WJ Terstappen LW Hayes DF Circulating tumor cells, disease progression, and survival in metastatic breast cancer N Engl J Med 2004 351 781 791 10.1056/NEJMoa040766 15317891

[B43] de Bono JS Scher HI Montgomery RB Parker C Miller MC Tissing H Doyle GV Terstappen LW Pienta KJ Raghavan D Circulating tumor cells predict survival benefit from treatment in metastatic castration-resistant prostate cancer Clin Cancer Res 2008 14 6302 6309 10.1158/1078-0432.CCR-08-0872 18829513

[B44] Cohen SJ Punt CJ Iannotti N Saidman BH Sabbath KD Gabrail NY Picus J Morse M Mitchell E Miller MC Doyle GV Tissing H Terstappen LW Meropol NJ Relationship of circulating tumor cells to tumor response, progression-free survival, and overall survival in patients with metastatic colorectal cancer J Clin Oncol 2008 26 3213 3221 10.1200/JCO.2007.15.8923 18591556

[B45] Rink M Chun FK Dahlem R Soave A Minner S Hansen J Stoupiec M Coith C Kluth LA Ahyai SA Friedrich MG Shariat SF Fisch M Pantel K Riethdorf S Prognostic role and HER2 expression of circulating tumor cells in peripheral blood of patients prior to radical cystectomy: a prospective study Eur Urol 2012 61 810 817 10.1016/j.eururo.2012.01.017 22277196

[B46] Naoe M Ogawa Y Morita J Omori K Takeshita K Shichijyo T Okumura T Igarashi A Yanaihara A Iwamoto S Fukagai T Miyazaki A Yoshida H Detection of circulating urothelial cancer cells in the blood using the Cell Search System Cancer 2007 109 1439 1445 10.1002/cncr.22543 17326057

[B47] Okumura Y Tanaka F Yoneda K Hashimoto M Takuwa T Kondo N Hasegawa S Circulating tumor cells in pulmonary venous blood of primary lung cancer patients Ann Thorac Surg 2009 87 1669 1675 10.1016/j.athoracsur.2009.03.073 19463575

[B48] Steen S Nemunaitis J Fisher T Kuhn J Circulating tumor cells in melanoma: a review of the literature and description of a novel technique Proc (Bayl Univ Med Cent) 2008 21 127 132 18382750 10.1080/08998280.2008.11928377 PMC2277345

[B49] Gradilone A Naso G Raimondi C Cortesi E Gandini O Vincenzi B Saltarelli R Chiapparino E Spremberg F Cristofanilli M Frati L Aglianò AM Gazzaniga P Circulating tumor cells (CTCs) in metastatic breast cancer (MBC): prognosis, drug resistance and phenotypic characterization Ann Oncol 2011 22 86 92 10.1093/annonc/mdq323 20603432

[B50] Pantel K Brakenhoff RH Brandt B Detection, clinical relevance and specific biological properties of disseminating tumour cells Nat Rev Cancer 2008 8 329 340 10.1038/nrc2375 18404148

[B51] Meng S Tripathy D Shete S Ashfaq R Saboorian H Haley B Frenkel E Euhus D Leitch M Osborne C Clifford E Perkins S Beitsch P Khan A Morrison L Herlyn D Terstappen LW Lane N Wang J Uhr J uPAR and HER-2 gene status in individual breast cancer cells from blood and tissues Proc Natl Acad Sci U S A 2006 103 17361 17365 10.1073/pnas.0608113103 17079488 PMC1838539

[B52] Maheswaran S Sequist LV Nagrath S Ulkus L Brannigan B Collura CV Inserra E Diederichs S Iafrate AJ Bell DW Digumarthy S Muzikansky A Irimia D Settleman J Tompkins RG Lynch TJ Toner M Haber DA Detection of mutations in EGFR in circulating lung-cancer cells N Engl J Med 2008 359 366 377 10.1056/NEJMoa0800668 18596266 PMC3551471

[B53] Bidard FC Mathiot C Degeorges A Etienne-Grimaldi MC Delva R Pivot X Veyret C Bergougnoux L de Cremoux P Milano G Pierga JY Clinical value of circulating endothelial cells and circulating tumor cells in metastatic breast cancer patients treated first line with bevacizumab and chemotherapy Ann Oncol 2010 21 1765 1771 10.1093/annonc/mdq052 20233745

[B54] Budd GT Cristofanilli M Ellis MJ Stopeck A Borden E Miller MC Matera J Repollet M Doyle GV Terstappen LW Hayes DF Circulating tumor cells versus imaging–predicting overall survival in metastatic breast cancer Clin Cancer Res 2006 12 6403 6409 10.1158/1078-0432.CCR-05-1769 17085652

[B55] Allan AL Keeney M Circulating tumor cell analysis: technical and statistical onsiderations for application to the clinic J Oncol 2010 2010 426218 20049168 10.1155/2010/426218 PMC2798617

[B56] Adams AA Okagbare PI Feng J Hupert ML Patterson D Göttert J McCarley RL Nikitopoulos D Murphy MC Soper SA Highly efficient circulating tumor cell isolation from whole blood and label-free enumeration using polymer-based microfluidics with an integrated conductivity sensor J Am Chem Soc 2008 130 8633 8641 10.1021/ja8015022 18557614 PMC2526315

[B57] Kirby BJ Jodari M Loftus MS Gakhar G Pratt ED Chanel-Vos C Gleghorn JP Santana SM Liu H Smith JP Navarro NV Tagawa ST Bander NH Nanus DM Giannakakou P Functional characterization of circulating tumor cells with a prostate-cancer-specific microfluidic device PLoS One 2012 7 e35976 Epub 32012 Apr 35927 10.1371/journal.pone.0035976 22558290 PMC3338784

[B58] Dancey JE Dobbin KK Groshen S Jessup JM Hruszkewycz AH Koehler M Parchment R Ratain MJ Shankar LK Stadler WM True LD Gravell A Grever MR; Biomarkers Task Force of the NCI Investigational Drug Steering Committee: Guidelines for the development and incorporation of biomarker studies in early clinical trials of novel agents Clin Cancer Res 2010 16 1745 1755 10.1158/1078-0432.CCR-09-2167 20215558

[B59] Allard WJ Matera J Miller MC Repollet M Connelly MC Rao C Tibbe AG Uhr JW Terstappen LW Tumor cells circulate in the peripheral blood of all major carcinomas but not in healthy subjects or patients with nonmalignant diseases Clin Cancer Res 2004 10 6897 6904 10.1158/1078-0432.CCR-04-0378 15501967

[B60] Meng S Tripathy D Frenkel EP Shete S Naftalis EZ Huth JF Beitsch PD Leitch M Hoover S Euhus D Haley B Morrison L Fleming TP Herlyn D Terstappen LW Fehm T Tucker TF Lane N Wang J Uhr JW Circulating tumor cells in patients with breast cancer dormancy Clin Cancer Res 2004 10 8152 8162 10.1158/1078-0432.CCR-04-1110 15623589

[B61] Smerage JB Doyle GV Budd GT Schott AF Blayney DW Wicha MS Repollet M Terstappen LW Hayes DF The detection of apoptosis and Bcl-2 expression in circulating tumor cells (CTCs) from women being treated for metastatic breast cancer Proc Am Assoc Cancer Res 2006 47 abstr 792

[B62] Taube SE Clark GM Dancey JE McShane LM Sigman CC Gutman SI A perspective on challenges and issues in biomarker development and drug and biomarker codevelopment J Natl Cancer Inst 2009 101 1453 1463 10.1093/jnci/djp334 19855077 PMC2773185

[B63] Lianidou ES Circulating tumor cells--new challenges ahead Clin Chem 2012 58 805 807 10.1373/clinchem.2011.180646 22353213

[B64] Hayes DF Smerage J Is there a role for circulating tumor cells in the management of breast cancer? Clin Cancer Res 2008 14 3646 3650 10.1158/1078-0432.CCR-07-4481 18559576

[B65] Reyal F Valet F de Cremoux P Mathiot C Decraene C Asselain B Brain E Delaloge S Giacchetti S Marty M Pierga JY Bidard FC Circulating tumor cell detection and transcriptomic profiles in early breast cancer patients Ann Oncol 2011 22 1458 1459 10.1093/annonc/mdr144 21525400

